# The Association of Ethnic Minority Density with Late Entry into Antenatal Care in the Netherlands

**DOI:** 10.1371/journal.pone.0122720

**Published:** 2015-04-09

**Authors:** Anke G. Posthumus, Vera L. N. Schölmerich, Eric A. P. Steegers, Ichiro Kawachi, Semiha Denktaş

**Affiliations:** 1 Erasmus University Medical Centre, Department of Obstetrics and Gynecology, Division of Obstetrics and Prenatal Medicine, Rotterdam, the Netherlands; 2 VU University Amsterdam, Department of Organization Sciences, Amsterdam, the Netherlands; 3 Harvard School of Public Health, Department of Social and Behavioral Sciences, Boston, Massachusetts, United States of America; Institute of Preventive Medicine, DENMARK

## Abstract

In the Netherlands, non-Western ethnic minority women make their first antenatal visit later than native Dutch women. Timely entry into antenatal care is important as it provides the opportunity for prenatal screening and the detection of risk factors for adverse pregnancy outcomes. In this study we explored whether women's timely entry is influenced by their neighborhood. Moreover, we assessed whether ethnic minority density (the proportion of ethnic minorities in a neighborhood) influences Western and non-Western ethnic minority women's chances of timely entry into care differently. We hypothesized that ethnic minority density has a protective effect against non-Western women's late entry into care. Data on time of entry into care and other individual-level characteristics were obtained from the Netherlands Perinatal Registry (2000-2008; 97% of all pregnancies). We derived neighborhood-level data from three other national databases. We included 1,137,741 pregnancies of women who started care under supervision of a community midwife in 3422 neighborhoods. Multi-level logistic regression was used to assess the associations of individual and neighborhood-level determinants with entry into antenatal care before and after 14 weeks of gestation. We found that neighborhood characteristics influence timely entry above and beyond individual characteristics. Ethnic minority density was associated with a higher risk of late entry into antenatal care. However, our analysis showed that for non-Western women, living in high ethnic minority density areas is less detrimental to their risk of late entry than for Western women. This means that a higher proportion of ethnic minority residents has a protective effect on non-Western women's chances of timely entry into care. Our results suggest that strategies to improve timely entry into care could seek to create change at the neighborhood level in order to target individuals likely of entering care too late.

## Introduction

International studies have shown that pregnant women from ethnic minority backgrounds tend to enter antenatal care at a significantly higher gestational age than ethnic majority women.[1, 2] Research in the Netherlands points in the same direction. Women from all non-Western ethnic minorities except those with a Turkish or Hindustani-Surinamese background make their first antenatal visit later than their Native Dutch counterparts. For example, multiparous Creole-Surinamese women entered antenatal care after 14 weeks of gestation in 49% of cases, against 11% for multiparous Dutch women.[3]

Late entry into antenatal care is problematic, as it is associated with a higher risk for adverse birth outcomes. These include abruptio placentae, chorioamnionitis, preterm birth, low birth weight and fetal and neonatal death.[4] Pregnant women in the Netherlands are advised to enter antenatal care between 8 and 10 weeks of gestation. If they enter care too late, i.e. after 14 weeks of gestation, they miss the opportunity to receive prenatal screening for a range of syndromes and congenital anomalies.[5]

Commonly cited risk factors in international studies for late entry into care are single status, young maternal age, poor language proficiency, maternal education of less than 5 years, multiparity, unplanned and unwanted pregnancy, difficulty in arranging an appointment for antenatal care, and being uninsured.[6] In the Netherlands, research has shown that a lack of knowledge of the Western healthcare system and poor language proficiency are important reasons for inadequate antenatal care usage among non-Western ethnic minority women.[7] While studies conducted in the Netherlands on time of entry into antenatal care have focused on these individual-level determinants, previous research in the United States and Canada found associations between the area of residence and timing of entry into antenatal care.[3, 8–10] Neighborhood characteristics may affect health outcomes over and beyond the influence of individual determinants. In other words, it is possible that certain neighborhoods are more or less conducive to pregnant women’s timing of entry into care.

In this study we explored the association between the proportion of non-Western ethnic minorities in a neighborhood (i.e. ethnic minority density) and late entry into antenatal care in the Netherlands. Moreover, we assessed whether ethnic minority density has a different influence on Western and non-Western ethnic minority women’s time of entry into care. We hypothesized that ethnic minority density has a protective effect on non-Western women’s timely entry into care. This is based on the findings of a recent Dutch study by Schölmerich *et al*. who found that while non-Western ethnic minorities generally have more adverse birth outcomes compared to Western women, this trend is reversed in areas with high ethnic minority density.[11] This means that while non-Western ethnicity is a risk factor at the individual-level, residence in a neighborhood of high ethnic minority density is a *protective* factor for non-Western women’s birth outcomes. A similar protective effect of high ethnic minority density has been found in studies on other health outcomes such as mental health and self-rated health and is known as the ‘ethnic density effect’.[11–13]

Studies have explored possible mechanisms underlying this ‘ethnic density effect’. One explanation is that ethnic minorities residing in neighborhoods with high ethnic minority density exhibit better health outcomes than ethnic majority groups because they experience higher levels of *bonding social capital*. Bonding social capital refers to ‘horizontal’ ties between members of a network who see themselves as similar (homogenous networks, such as ethnic groups).[14, 15] Social capital has been conceptualized to influence health in several different ways—plausibly, these patterns also apply to *bonding* social capital: firstly, by promoting the exchange of resources between residents, secondly by residents engaging in collective action to improve access to local services and amenities, thirdly through social control over healthy behavior, and lastly by more efficient diffusion of health related information.[16, 17]

While social capital is generally seen as having a positive influence on health (behaviors), studies have found that bonding social capital may promote health but may also act as a source of strain (and hence a detriment to health) in resource-poor settings.[18] Scholars have explained this phenomenon via two pathways. For one, bonding social capital may facilitate good health through the exchange of resources between neighbors, but high reliance on mutual exchange of reciprocity can result in excessive obligations placed upon residents to help each other, which might be detrimental to health. In addition, while bonding social capital can assist in the diffusion of information, the closed nature of social ties in such communities can also restrict the flow of information from the outside (e.g. new information about changes in the Dutch obstetric system) and maintain the circulation of unreliable information.[19] This could lead to less timely and adequate use of antenatal care. This means that neighborhood ethnic minority density could either have a detrimental or beneficial effect on utilization of antenatal care.

As we assume that the neighborhood characteristic ethnic minority density is a proxy for bonding social capital for *non-Western* residents, we also wanted to control for bonding social capital of *Western* residents. Based on a recent study by Schölmerich *et al*, we use a measurement of neighborhood social capital for this proxy.[11] This measurement was derived from a nationally representative data set, of which 82.7% of the respondents were Western. Furthermore, we control for the following other neighborhood characteristics: feeling of safety, socio-economic status, level of urbanity and home maintenance. We included these variables because prior studies in the Netherlands found an association between them and adverse birth outcomes as well as general health.[11, 20, 21] The causal pathways between neighborhood influences on (prenatal) health have not been completely unraveled and may be mediated by adverse health behaviors such as late entry into care.

The objective of our study was to explore the independent association between neighborhood ethnic minority density and late entry into antenatal care in the Netherlands. Moreover we wanted to investigate whether neighborhood ethnic minority density affects Western and non-Western women differently. We hypothesize that in line with the study by Schölmerich *et al*., ethnic minority density will have a beneficial effect on time of entry into care for non-Western women when compared to Western women.[11]

## Data & Methods

To determine the association between ethnic minority density and the risk of late entry into antenatal care in the Netherlands, we extracted neighborhood-level variables from three national datasets, and linked this with a large dataset on individual pregnancy cases using the four-digit zip code for neighborhoods.

### Ethics and consent

The use of the anonymized patient data for this study was approved by the Netherlands Perinatal Registry (project number 13.50) (additional information on the registry: www.perinatreg.nl/home_english). Written consent from pregnant women was not needed as the registry protects their anonymity.

### Outcome variable

Timely entry into care was defined as entry at any time before 14 weeks of gestation; late entry into care was defined as starting after 14 weeks of gestation (0 = not late, 1 = late). The cut-off point of after 14 weeks of gestation was chosen because entry into care after 14 weeks of gestation excludes a woman from prenatal screening on Down, Edwards and Patau syndrome in the Netherlands and early detection and modification of other medical and non-medical risk factors (such as illicit drug use) for adverse pregnancy outcome.[5, 22]

### Individual level determinants

The data on entry into care were acquired from the Netherlands Perinatal Registry, which contains 97% of Dutch pregnancies since the year 2000. Midwives, gynecologists and neonatologists supply these data. Validation studies comparing the data from the Perinatal Registry and Statistics Netherlands (national statistics bureau [23]) have shown that underreporting of information by practitioners for the Perinatal Registry is negligible. However no specific validation has taken place for the data on time of entry into care. We will further elaborate on this in the discussion section.

For this study data we selected singleton pregnancies in the datasets from 2000 up to and including 2008, because then both individual (i.e. pregnancy cases) and neighborhood level data are derived from approximately the same time frame. The weeks of gestation at entry into care were regrouped dichotomously into ‘up to and including 14 weeks of gestation’ and ‘after 14 weeks of gestation’. Based on previous studies on the association of maternal covariates and time of entry in to care, we included the following maternal covariates: maternal age, parity and ethnicity.

In the Netherlands Perinatal Registry, ethnicity is divided into the following categories: Western Dutch, Western Other, Mediterranean, Asian, African, South Asian, or other non-Western. Most non-Western immigrants in the Netherlands are from Turkey, Morocco, Surinam and the Dutch Antilles. The recording of ethnicity in the Netherlands Perinatal Registry is challenging for two reasons: 1) Maternal ethnicity is based on either self-declared ethnicity or country of birth of the mother or her parents causing heterogeneity in registration; 2) the categorization in the registry is not in line with international classifications, making comparisons difficult. Therefore we dichotomized ethnicity into being from ‘Western’ or ‘non-Western’ descent for the purpose of this study.

### Neighborhood level determinants

Four-digit zip code areas were used to define neighborhoods. In 2006 the four-digit zip code neighborhoods had—on average—4080 residents. This makes the neighborhoods comparable to Lower Layer Super Output Areas in the United Kingdom or census tracts in the United States. Because neighborhoods in the Netherlands are sufficiently uniform in terms of their socio-cultural characteristics, the four-digit zip code areas are adequate units for contextual investigation. [24] Data on the neighborhood characteristics were obtained from Statistics Netherlands, the Housing & Living Survey and the Netherlands Institute for Social Research.[23, 25, 26] These data were collected between 2005 and 2006.

As mentioned in the introduction, we included six neighborhood characteristics in our analysis. A more detailed description of the characteristics is given in Table 1. All neighborhood characteristics were recoded into z-scores. The characteristics were constructed using the same aggregation techniques and data sets that Schölmerich *et al*. and Mohnen *et al*. applied in their respective studies.[11, 20].

**Table 1:** Detailed description of neighborhood level variables included in the multilevel model.

**Variable Name**	**Meaning**	**Measurement**	**Source**	**Ref.**
Ethnic minority density	Concentration of people from ethnic minorities	% of residents from non-Western ethnic backgrounds per 4 digit zip code. Non-Western ethnicity is defined as an individual or at least one of the individual’s parents originating from Africa, Latin America, Asia (except Indonesia and Japan) or Turkey. Higher values indicate a higher concentration of ethnic minorities	Statistics Netherlands	[26]
Social capital	Access to resources that are generated by relationships between residents in a tightly knit and cohesive community	Five-point Likert scale (I totally agree—I totally do not agree). 1) Contact with direct neighbors; 2) Contact with other neighbors; 3) Whether people in the neighborhood know each other; 4) Whether neighbors are friendly to each other; 5) Whether there is a friendly and sociable atmosphere in the neighborhood. Social capital scores which were created using an ‘ecometrics’ procedure were provided by Schölmerich *et al*. The reliability of the scale was acceptable, with an estimator of 0.595 (in accordance with Hox). Higher values indicate higher levels of social capital	House and Living Survey (items) Schölmerich *et al*. (Ecometrics score)	[11, 25]
Feeling of safety	Perception of safety in the neighborhood	Five-point Likert scale (I totally agree—I totally do not agree). Statement: “I am scared of being harassed or assaulted in this neighborhood" Higher values indicate higher levels of perceived safety	House and Living Survey	[25]
Socio-economic status	A group's position within a hierarchical social structure	Average income, % of people with low income, % of people with a low education and % of unemployed people in a neighborhood. Higher values indicate a higher socioeconomic status.	Netherlands Institute for Social Research	[23]
Level of urbanity of the neighborhood	Degree of urbanity of the municipality a neighborhood is situated in	Number of addresses per square kilometer (km2). 1) Rural, up to 499 addresses per km2; 2) Semi-rural, 500–999 addresses per km2; 3) Intermediate urban-rural, 1000–1499 addresses per km2; 4) Semi-urban, 1500–2499 addresses per km2; 5) Urban, more than 2499 addresses per km2. Higher values indicate higher levels of urbanity.	House and Living Survey	[25]
Home maintenance	Proxy for the environmental condition in a neighborhood	Five-point Likert scale (I totally agree—I totally do not agree). Question: “Is your house in bad condition?” Higher values indicate better home maintenance.	House and Living Survey	[25]

In this study only women who started care with a community midwife (the ‘first tier’) were included. The Dutch obstetric care system consists of three ‘tiers’. The first tier consists of autonomously working community midwives who take care of low risk women.[27] When complications (threaten to) occur, women are referred to the second tier of care, consisting of obstetricians in hospitals. The third tier of care consists of academic obstetric care. A quarter of women enter obstetric care immediately in the second or third tier because of medical risks or complications at the start of their pregnancy.[28] We focused on the first tier population because immediate entry into care in the second and third tier is above all determined by the patients’ medical and obstetric history. Typically these women have previously received explicit instructions about their antenatal care and the importance of timely entry. The women included in this study—the first tier population—form the greatest portion of all pregnant women in the Netherlands, namely 74%. These women are not just a low risk population because many of them will be referred to the second tier of care, either during pregnancy, labor or the postpartum period because of new risks or complications.[29] This means that the women included in this study are still heterogeneous in terms of their risk profile, making comparison to other studies possible.

**Fig 1:**
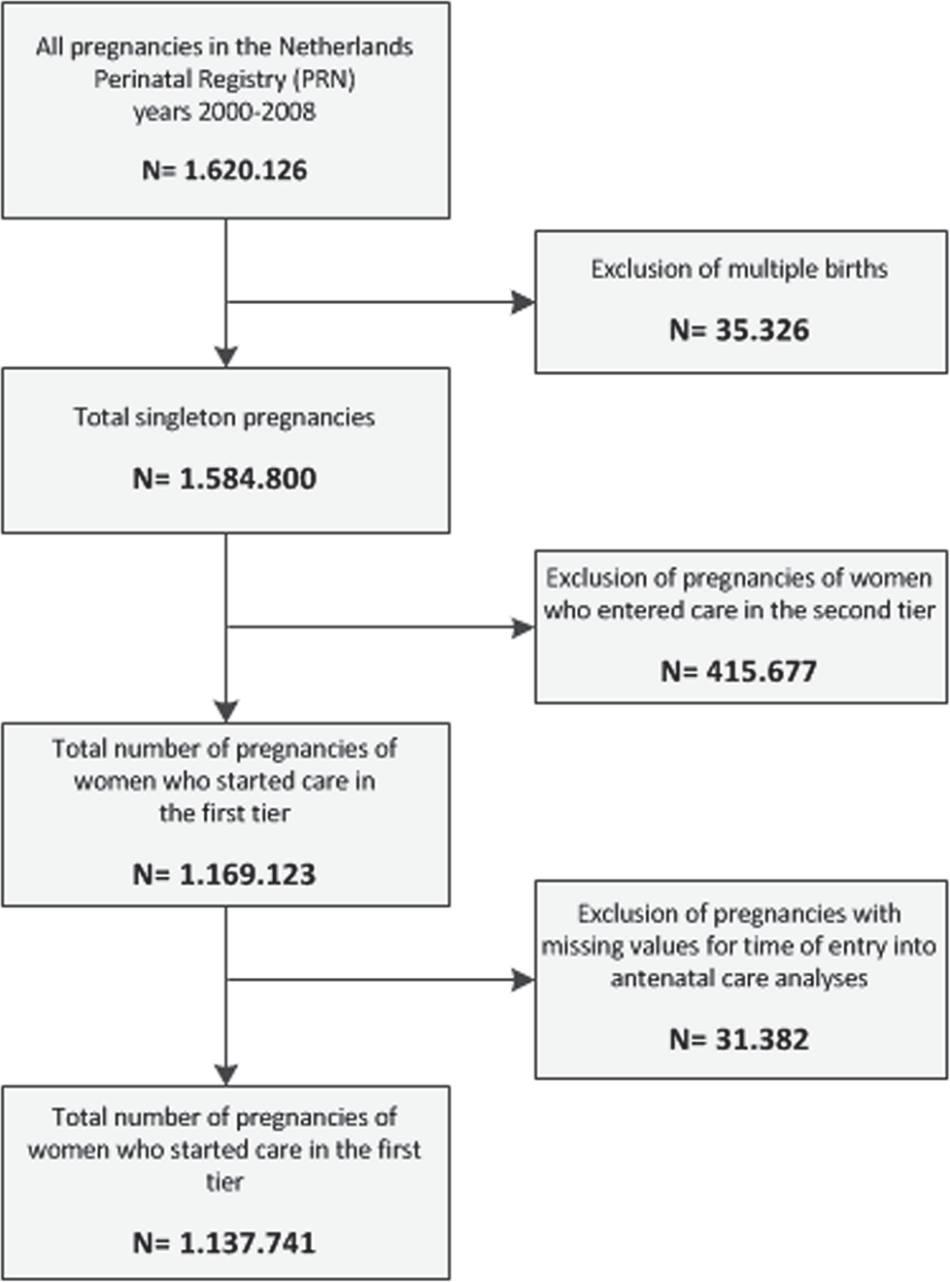
Exclusion of pregnancies. This Fig. shows the number of pregnancies excluded from the multilevel logistic regression analysis.

The final analysis included 1,137,741 pregnancies and 3.422 neighborhoods. 35,326 (2.2%) pregnancies were excluded because they were multiple pregnancies and 31,382 (1.9%) pregnancies because individual or neighborhood characteristics were missing. Non-Western women were slightly more likely to have missing values than non-Western women (2.8% vs 2.7%). 580 neighborhoods (14% of the total) were excluded because not all six neighborhood characteristics were available (Fig 1). Most of the excluded neighborhoods had too few inhabitants to be included in the study because they were rural or industrial areas.

### Analytical strategy

Because of the hierarchical nature of the data, in which pregnant women (level 1) are nested within neighborhoods (level 2) we performed multilevel logistic regression analyses. First, to determine whether clustering was present we fitted a “null-model” which only contained a random intercept.To establish the presence of clustering we calculated the intra-class correlation (ICC), using the following formula in which sigma-squared is the intercept variance:[30]

$$ICC=\frac{\sigma^{2}}{\sigma^{2}+3.29^{,}}$$

The ICC can range from 0 to 1. When it deviates from zero, it is appropriate to use multilevel analyses.[31] We found an ICC of 9.5% (results not shown) justifying this modelling approach. The null-model was then expanded to include the individual level characteristics age, parity and ethnicity as fixed effects to examine the influence of these on late entry into care (model 1). Thereafter, we separately added the neighborhood contextual variables ‘neighborhood social capital’ and ‘ethnic density’ (model 2 and 3) to investigate their specific influence, before adding the other neighborhood variables (model 4). Consecutively an interaction term was included for non-Western ethnicity*neighborhood social capital (model 5) and non-Western ethnicity* ethnic minority density (model 6) to investigate the potential difference in impact of these neighborhood characteristics on Western and non-Western women. Although we were primarily interested in the interaction for non-Western ethnicity*ethnic minority density, we also tested the interaction with neighborhood social capital because Schölmerich *et al*. found this interaction term to be significant in their analysis of birth outcomes in the Netherlands.[11] In the final model, all individual variables, neighborhood contextual variables and interaction terms were included (model 7). All analyses were performed using SPSS version 20.

## Results

Between 2000 and 2008 the prevalence of entry into antenatal care after 14 weeks of gestation was 17.9%. Table 2 shows the demographic characteristics of the study population and Table 3 shows the descriptive statistics of the neighborhoods. The correlations between the neighborhood variables are presented in S1 Appendix. Most importantly, neighborhoods with higher socio-economic status generally had lower ethnic minority density (corr. -0.56, p<0.001).

**Table 2:** Descriptive statistics of individual variables and time of entry into care.

	**Total**		**Western**		**Non-Western**		**Significance**
** **	**N**	**(%)**	**N**	**(%)**	**N**	**(%)**	**(p-value)**
**Total singelton births—first tier of care**	1,137,741 (100)	(100.0)	959,771	(84.4)	177,970	(15.6)	
**Maternal age**							<0.01
<25 years	141,239	(12.4)	93,239	(9.7)	48,000	(27.0)	
25–29 years	343,101	(30.2)	285,336	(29.7)	57,765	(32.5)	
30–34 years	451,282	(39.7)	403,838	(42.1)	47,444	(26.7)	
35–39 years	181,309	(15.9)	160,403	(16.7)	20,906	(11.7)	
>40 years	20,810	(1.8)	16,955	(1.8)	3,855	(2.2)	
**Parity**							<0.01
Primiparous (first birth)	541,117	(47.6)	465,825	(48.5)	75,292	(42.3)	
Multiparous (second or higher birth)	596,624	(52.4)	493,946	(51.5)	102,678	(57.7)	
**Time of entry into care**							<0.01
< 14 weeks of gestation	934,453	(82.1)	820,752	(85.5)	113,701	(63.9)	
≥ 14 weeks of gestation	203,288	(17.9)	139,019	(14.5)	64,269	(36.1)	

(Source: Perinatal Registration Netherlands, 2000–2008).

**Table 3:** Descriptive statistics of the neighborhoods.

**Neighborhood characteristic**	**Low (%)**	**Medium (%)**	**High (%)**
Ethnic minority density^1^	91.8	8.1	0.2
Neighborhood social capital^2^	3.7	81.2	15.1
Socioeconomic status^3^	20.3	63.4	16.3
Feeling of safety^4^	1.4	22.1	77.4
Level of urbanity^5^	50.2	19.4	30.5
Home maintenance^6^	1.4	33.3	65.3

The figures presented in this table are crude proportions. For the purpose of our analyses we transformed these into Z-scores. The median number of deliveries per neighborhood was 349 (range: 56–602, 20^th^-80^th^ percentile).

^1^ Low: <20%; medium: 20–80%; high >80% inhabitants from non-Western origin.

^2^ Low: <3 on the 5-point Likert scale; medium: 3 on the 5-point Likert scale; high: >3 on the 5-point Likert scale.

^3^ Low: <20^th^ percentile; medium: 20-80^th^ percentile; high >80^th^ percentile.

^4^ Statement: “I am scared of being harassed or assaulted in this neighborhood.” Low: on average inhabitants agree; Medium: on average inhabitants don’t agree and don’t disagree; high: on average inhabitants don’t agree.

^5^ Low: <1000 addresses per km2; medium: 1000–1500 addresses per km2; high: >1500 addresses per km2.

^6^ Question: “Is your house in bad condition?” Low: on average inhabitants agree; Medium: on average inhabitants don’t agree and don’t disagree; high: on average inhabitants don’t agree.

Table 4 / model 1 shows the odds ratios for the individual level characteristics in our logistic regression analysis with ‘late entry into care’ as the outcome variable. Women in the age category of 30 to 35 years were most likely to enter antenatal care late. Non-Western ethnicity was also strongly associated with higher risk for late entry into care. Contrarily, we found no significant association of parity and time of entry into care. Moreover, the estimates for all of these individual level variables showed minimal change across the models.

**Table 4:** Multilevel logistic regression models of ethnic minority density and other individual and neighborhood characteristics on late entry into care (after 14 weeks of gestation).

**n = 1,137,741 nb = 3422**		**Model 1**		**Model 2**		**Model 3**		**Model 4**		**Model 5**		**Model 6**		**Model 7**	
Intercept		0.15	(0.15/ 0.15)***	0.15	(0.15/ 0.15) ***	0.15	(0.15/ 0.15) ***	0.15	(0.15/ 0.15) ***	0.15	(0.15/ 0.15) ***	0.15	(0.15/ 0.15) ***	0.15	(0.15/ 0.15) ***
**Individual level**		** **		** **		** **		** **		** **		** **		** **	
Maternal age (Ref. = 25–29 yrs)	*<25yr*	1.80	(1.77/ 1.83) ***	1.80	(1.77/ 1.83) ***	1.80	(1.77/ 1.83) ***	1.80	(1.77/ 1.83) ***	1.80	(1.77/ 1.82) ***	1.79	(1.77/ 1.82) ***	1.79	(1.77/ 1.82) ***
	*30-34yr*	3.06	(2.96/ 3.15) ***	3.05	(2.96/ 3.15) ***	3.05	(2.96/ 3.15) ***	3.06	(2.97/ 3.16) ***	3.06	(2.96/ 3.15) ***	3.05	(2.96/ 3.15) ***	3.05	(2.96/ 3.15) ***
	*35-39yr*	1.06	(1.05/ 1.07) ***	1.06	(1.05/ 1.07) ***	1.06	(1.05/ 1.07) ***	1.06	(1.05/ 1.07) ***	1.06	(1.05/ 1.07) ***	1.06	(1.05/ 1.07) ***	1.06	(1.05/ 1.07) ***
	*>40yr*	1.56	(1.54/ 1.59) ***	1.56	(1.54/ 1.59) ***	1.56	(1.54/ 1.59) ***	1.57	(1.54/ 1.59) ***	1.56	(1.54/ 1.59) ***	1.56	(1.54/ 1.59) ***	1.56	(1.54/ 1.59) ***
Parity (Ref. = multiparous)	*Primi-parous*	1.00	(0.99/1.01)	1.00	(0.99/ 1.01)	1.00	(0.99/ 1.01)	1.00	(0.99/ 1.01)	1.00	(0.99/ 1.01)	0.99	(0.98/ 1.01)	0.99	(0.98/ 1.01)
Ethnicity (Ref. = Western)	*non-Western*	2.63	(2.60/ 2.67) ***	2.62	(2.59/ 2.66) ***	2.62	(2.58/ 2.65) ***	2.61	(2.58/ 2.65) ***	2.68	(2.64/ 2.72) ***	2.72	(2.68/ 2.77) ***	2.73	(2.69/ 2.77) ***
**Neighborhood level **															
Ethnic minority density						1.07	(1.04/ 1.09) ***.09) ***	1.16	(1.11/ 1.20) ***	1.16	(1.12/ 1.21) ***	1.21	(1.16/ 1.26) ***	1.21	(1.16/ 1.26) ***
Neighborhood social capital				1.00	(0.97/ 1.02)			1.01	(0.98/ 1.04)	1.00	(0.97/ 1.03)	1.02	(0.99/ 1.04)	1.01	(0.99/ 1.04)
Socio-economic status								0.97	(0.95/ 1.00) *	0.97	(0.95/ 1.00) *	0.97	(0.95/ 1.00) *	0.97	(0.95/ 1.00) *
Level of urbanity								0.88	(0.86/ 0.90) ***	0.88	(0.86/ 0.90) ***	0.87	(0.85/ 0.90) ***	0.87	(0.85/ 0.90) ***
Home maintenance								0.97	(0.95/ 0.98) ***	0.97	(0.95/ 0.99) ***	0.97	(0.95/ 0.99) ***	0.97	(0.95/ 0.99)**
Feeling of safety								1.00	(0.98/ 1.02)	1.00	(0.98/ 1.02)	1.00	(0.98/ 1.02)	1.00	(0.98/ 1.02)
Neighb. social capital*non-Western										1.06	(1.05/ 1.08) ***			1.01	(1.00/ 1.03)
Ethnic minority density*non-Western												0.97	(0.96/ 0.98) ***	0.93	(0.91/ 0.94) ***

(Odds ratios, 95% confidence intervals in parentheses).

*n = number of pregnancies; nb = number of neighborhoods; significance (p-value)*:

*p≤0.*05*

**p≤0.*01*

***p≤0.*001*.

**Fig 2:**
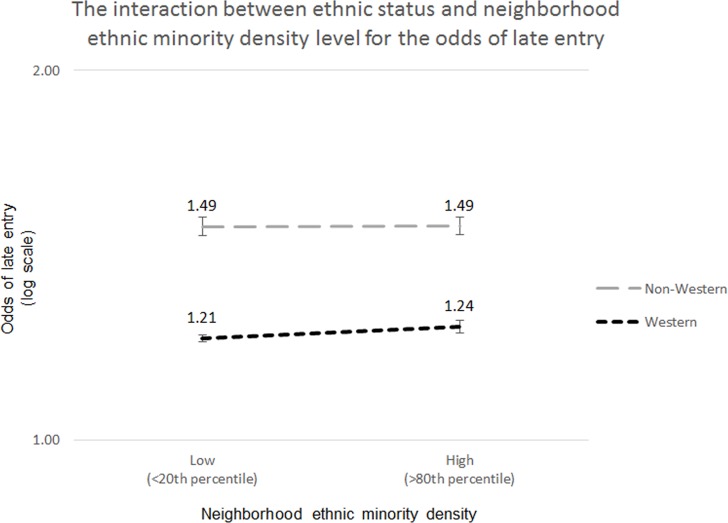
The interaction between ethnic status and neighborhood ethnic minority density level for the odds of late entry. This Fig. demonstrates that higher proportions of ethnic minority density in a neighborhood have a less detrimental effect on non-Western women than on Western women in terms of their risk of late entry into care. Low ethnic density: 20 percent neighborhoods with the lowest proportions of non-Western inhabitants; high ethnic density: 20 percent neighborhoods with the highest proportions of non-Western inhabitants.

In model 2 of our analysis, neighborhood social capital was added (Table 4). The association of this variable with late entry into care was not significant and remained so in all other models. In contrast, ethnic minority density (model 3) was significantly associated with late entry into care. This effect remained present after controlling for the other neighborhood contextual variables in model 4. Though feeling of safety had no effect, higher levels of socioeconomic status, home maintenance and level of urbanity were associated with lower risks of late entry into care. The latter showed the most notable effect of these three. Model 5 and 6 include the interaction terms for neighborhood social capital*non-Western ethnicity and ethnic minority density*non-Western ethnicity, respectively. Though the interaction term for neighborhood social capital*non-Western showed a significant effect in model 5, this effect was no longer present in the full model (model 7). However, the interaction term for ethnic minority density*non-Western ethnicity did remain significant in the full model. From this follows that ethnic minority density is associated with 1.21 times the odds of late entry into care for Western women and 1.13 times the odds for non-Western women (calculated: exp (ln(1.21) + ln(0.93)). In the full model, again neighborhood level of urbanity showed the most notable association (OR 0.87, 95%CI 0.85–0.90, p≤0.01). Fig 2 illustrates the interaction effect between ethnic status (non-Western or Western) and the level of ethnic minority density in a neighborhood for risk of late entry into care.

## Discussion

We found that neighborhood contexts influence timing of entry into antenatal care in the Netherlands. In particular, higher rates of neighborhood ethnic minority density are associated with a higher risk of late entry into antenatal care in the Netherlands. However, our analysis also shows that for non-Western women, living in high ethnic minority density areas is less detrimental to their timing of antenatal care than for Western women.

Similar to our study, Heaman *et al*. reported higher risks of inadequate antenatal care use for women living in neighborhoods with higher numbers of residents with an indigenous minority background.[9] It should be noted that inadequate use of care, the outcome measure of their study, was broader than our outcome measure. Inadequacy of care entails late entry into care and / or an insufficient number of antenatal appointments. There is no international consensus on the appropriate number of antenatal visits. Nevertheless, inadequacy of antenatal care is used in a number of studies because it is believed that it may be associated with adverse pregnancy outcomes.[1, 32–34] We were unable to analyze the number of antenatal visits each woman had because this is not recorded in the Netherlands Perinatal Registry. However, a systematic review by Feijen *et al*. showed that both late entry and an insufficient number of antenatal appointments share the same set of risk factors.[6] Therefore we believe that it is valid to compare our results with other studies focusing on inadequate use of antenatal care.

In line with previous studies, being of non-Western ethnic descent was amongst the most important predictors for late entry into care. This supports the commonly held view that ‘ethnicity’ (meaning a non-Western ethnic minority status) is a risk factor for health behavior, including adequate use of care.[35, 36] However, our analysis shows that for non-Western women, living in high ethnic minority density areas is less detrimental to their risk of late entry into antenatal care than for Western women. This means that while ethnic minority status is indeed not a protective factor in and of itself at the *individual* level, it seems to act as a protective factor for time of entry into care at the *neighborhood* level in areas where ethnic minorities are in the majority. Our results are in line with a recent study by Schölmerich *et al*. who found the same pattern for birth outcomes.[11] Similar to our study, Cubbin and colleagues found that place of residence influences ethnic minority and majority groups differently in terms of their risk for late entry into antenatal care.[37] The results were stratified for neighborhood deprivation levels instead of neighborhood ethnic minority density levels. But prior research—as well as our study (see S1 Appendix)—has shown a relation between higher levels of neighborhood deprivation and higher levels of ethnic minority density.[38] Cubbin and colleagues found that African American women in the least deprived areas (and presumably areas of lower ethnic minority density) were at higher risk of delayed entry into antenatal care than African American women living in moderately deprived areas. Contrastingly, in the most deprived areas (and presumably areas of higher ethnic minority density) the risk of late / no initiation of antenatal care was only elevated among European American women.

Various studies have suggested that for ethnic minority groups, ethnic minority density could be seen as a proxy for bonding social capital.[11, 39, 40] Applied to our study, this would mean that the non-Western women in our study have higher levels of bonding social capital than their Western counterparts in areas with high ethnic minority density. As outlined in the introduction, higher levels of *bonding* social capital have been associated with both *higher and* lower risk of adequate health care use. The findings from our study suggest that *bonding* social capital has a positive effect on time of entry into care of non-Western women. For these women, bonding social capital might enhance the chances of timely entry into care: firstly by promoting the exchange of resources between residents (for example money to take public transport to an antenatal care provider); secondly by having residents engage in collective action to improve access to local antenatal services; thirdly through social control over healthy behavior (in this case on timely entry into antenatal care); and lastly by more efficient diffusion of health related information (on the importance of timely entry into care, and access to antenatal care).[16, 17]

Neighborhood social capital showed no effect in our analysis. This was an unexpected finding. In the literature higher levels of neighborhood social capital are associated with more adequate use of care of Western women. [41] Moreover, based on a recent Dutch study on birth outcomes we had expected that this variable would act as a proxy for bonding social capital of Western residents. Our observations suggest that if Western women have access to bonding social capital, it does not protect them from late entry into care. In contrast, non-Western women benefit from their access to bonding social capital in terms of protection from late entry into care.

In line with previous studies in the Netherlands on other neighborhood effects, we found that home maintenance (reflecting the environmental conditions in a neighborhood) and level of urbanity were associated with slightly better outcomes.[11, 20] Similarly, Larson *et al*. reported that living in rural areas was strongly associated with late entry into antenatal care in the United States.[42] An explanation mentioned in this study that could also be plausible for our setting is longer travelling distances to care providers in rural areas. Lower neighborhood socioeconomic status was associated with a higher risk of late entry into care in all of our models. Two previous studies also reported that lower neighborhood socioeconomic status was associated with inadequate use of antenatal care.[9, 10]

## Strengths and Limitations

This study has a number of strengths and limitations that merit discussion. An important strength of our study was that it was conducted with a national dataset, with high coverage (97%) and a large number of participants (n = 1,137,741). Second, in our analyses we used appropriate and sophisticated techniques (multi-level analyses) to account for the clustering of women within neighborhoods. Our study should also be viewed in the light of its limitations. Due to the retrospective nature of the data no inferences could be made about causation, only about associations. The use of a dichotomous variable for ethnicity is both a strength and a weakness. As described in the methods section, it is less misclassified than the multiple categories in the Dutch Perinatal Registry. Yet, collapsing ethnicity into a dichotomous variable leads to grouping women together from heterogeneous backgrounds and with different health behaviors. Therefore the identification of different underlying mechanisms for different ethnic groups is not possible within this study. Moreover, in this study we did not have information on the migrant status of women. Although non-Western ethnicity is often associated with language barriers and lower health literacy levels, time spent in the ‘host’ country and the degree of acculturation influence health care behavior.[43, 44] Despite the lack of data on ethnic groups and migrant status, we hope to have shown with our study that ‘ethnicity’ can be beneficial and is not merely a risk factor.

As described in the methods section, the data on time of entry into antenatal care in the Netherlands Perinatal Registry has not been validated. In our data set, 17.2% of pregnancy cases were registered as late entry into care. This is comparable to a large Dutch cohort study—the Rotterdam-based Generation R study, which registered 19.8% of cases as entry into care after 14 weeks of gestation.[3]

The Netherlands Perinatal Registry database only contains information on individual births. Therefore we were unable to account for clustering of births within mothers. It is conceivable that mothers repeated their health care behavior (that is: time of entering care) across their consecutive pregnancies. Moreover, we were not able to control for certain maternal factors that have been associated with late entry into care in previous studies, such as an unwanted pregnancy, illicit drug use, individual socioeconomic status, level of education and language proficiency.[6, 7] Research in an urban group of Dutch pregnant women showed that 0.5% of them continued using illicit drugs throughout pregnancy.[22] A little less than six percent of pregnancies in the Netherlands are unwanted, of which only a part is carried to term.[45] Based on these Figs., unwanted pregnancies and illicit drug use are only present in a small portion of the population and are therefore less likely to have an important impact on our findings.

Lastly, we could not take other neighborhood characteristics into account that may also have influenced timing of entry into antenatal care in our study. Prior studies have demonstrated that quality of public transport and the density and accessibility of care facilities in neighborhoods influence timing of entry into care.[7, 10, 46]

## Future Recommendations

This study shows that place of residence and ethnic background matter for antenatal health care use in the Netherlands. Future research could concentrate on teasing apart the beneficial mechanisms within areas of high ethnic minority density leading to early entry into care (e.g. information sharing, financial support or other factors). Moreover, our results suggest that strategies to improve timely entry into care could seek to create change at the neighborhood level (e.g. increase social bonding) in order to target individuals likely of entering care too late. Also the relative disadvantage of Western women living in areas of high ethnic density needs to be considered, interventions should also focus on Western women living in these areas.

## Supporting Information

S1 Appendix Correlations of neighborhood variables included in the analysis. (DOCX)
