# Supplementary material for: The Association of Ethnic Minority Density with Late Entry into Antenatal Care in the Netherlands
Source: PLoS One. 2015 Apr 9;10(4):e0122720. doi: 10.1371/journal.pone.0122720 (PMC4391847; doi:10.1371/journal.pone.0122720)
Supplement: S1 Appendix — (DOCX) [file pone.0122720.s001.docx]

**Appendix 1:** Correlations of neighborhood variables included in the analyses

| **Neighborhood variables** | **1** | **2** | **3** | **4** | **5** | **6** |
| --- | --- | --- | --- | --- | --- | --- |
| 1. Ethnic minority density | 1 | - | - | - | - | - |
| 2. Neighborhood social capital | -,565^**^ | 1 | - | - | - | - |
| 3. Socio-economic status | -,562^**^ | ,346^**^ | 1 | - | - | - |
| 4. Urbanity | ,588^**^ | -,505^**^ | -,237^**^ | 1 | - | - |
| 5. Home maintenance | -,281^**^ | ,278^**^ | ,323^**^ | -,183^**^ | 1 | - |
| 6. Feeling of safety | -,412^**^ | ,385^**^ | ,293^**^ | -,320^**^ | ,264^**^ | 1 |

3,422 neighborhoods were included in the analysis.

*p≤0.05, **p≤0.01, ***p≤0.001
